# Supplementary material for: Lesbian and Gay Population, Work Experience, and Well-Being: A Ten-Year Systematic Review
Source: Int J Environ Res Public Health. 2024 Oct 14;21(10):1355. doi: 10.3390/ijerph21101355 (PMC11507352; doi:10.3390/ijerph21101355)
Supplement: Supplementary file 1 [file ijerph-21-01355-s001.zip › ijerph-3213948-supplementary.pdf]

Tab. S1. PRISMA 2020 Checklist

Section and Topic	Item #	Checklist item	Location where item is reported
**TITLE**			
Title	1	Identify the report as a systematic review.	p.1
**ABSTRACT**			
Abstract	2	See the PRISMA 2020 for Abstracts checklist.	p.1
**INTRODUCTION**			
Rationale	3	Describe the rationale for the review in the context of existing knowledge.	p.1-3
Objectives	4	Provide an explicit statement of the objective(s) or question(s) the review addresses.	p.3
**METHODS**			
Eligibility criteria	5	Specify the inclusion and exclusion criteria for the review and how studies were grouped for the syntheses.	p.3-4
Information sources	6	Specify all databases, registers, websites, organisations, reference lists and other sources searched or consulted to identify studies. Specify the date when each source was last searched or consulted.	p.3
Search strategy	7	Present the full search strategies for all databases, registers and websites, including any filters and limits used.	p.3-4
Selection process	8	Specify the methods used to decide whether a study met the inclusion criteria of the review, including how many reviewers screened each record and each report retrieved, whether they worked independently, and if applicable, details of automation tools used in the process.	p.4
Data collection process	9	Specify the methods used to collect data from reports, including how many reviewers collected data from each report, whether they worked independently, any processes for obtaining or confirming data from study investigators, and if applicable, details of automation tools used in the process.	p.4
Data items	10a	List and define all outcomes for which data were sought. Specify whether all results that were compatible with each outcome domain in each study were sought (e.g. for all measures, time points, analyses), and if not, the methods used to decide which results to collect.	p.4
	10b	List and define all other variables for which data were sought (e.g. participant and intervention characteristics, funding sources). Describe any assumptions made about any missing or unclear information.	p.4
Study risk of bias assessment	11	Specify the methods used to assess risk of bias in the included studies, including details of the tool(s) used, how many reviewers assessed each study and whether they worked independently, and if applicable, details of automation tools used in the process.	p.4
Effect measures	12	Specify for each outcome the effect measure(s) (e.g. risk ratio, mean difference) used in the synthesis or presentation of results.	NA
Synthesis methods	13a	Describe the processes used to decide which studies were eligible for each synthesis (e.g. tabulating the study intervention characteristics and comparing against the planned groups for each synthesis (item #5)).	p.3-4
	13b	Describe any methods required to prepare the data for presentation or synthesis, such as handling of missing summary statistics, or data conversions.	.p.3-4
	13c	Describe any methods used to tabulate or visually display results of individual studies and syntheses.	p. 4
	13d	Describe any methods used to synthesize results and provide a rationale for the choice(s). If meta-analysis was performed, describe the model(s), method(s) to identify the presence and extent of statistical heterogeneity, and software package(s) used.	p.4
	13e	Describe any methods used to explore possible causes of heterogeneity among study results (e.g. subgroup analysis, meta-regression).	p.4
	13f	Describe any sensitivity analyses conducted to assess robustness of the synthesized results.	p.4
Reporting bias assessment	14	Describe any methods used to assess risk of bias due to missing results in a synthesis (arising from reporting biases).	p.4
Certainty assessment	15	Describe any methods used to assess certainty (or confidence) in the body of evidence for an outcome.	p.4
**RESULTS**			
Study selection	16a	Describe the results of the search and selection process, from the number of records identified in the search to the number of studies included in the review, ideally using a flow diagram.	p.4

Tab. S1. PRISMA 2020 Checklist

Section and Topic	Item #	Checklist item	Location where item is reported
	16b	Cite studies that might appear to meet the inclusion criteria, but which were excluded, and explain why they were excluded.	Figure 1
Study characteristics	17	Cite each included study and present its characteristics.	p. 5-12
Risk of bias in studies	18	Present assessments of risk of bias for each included study.	p.4
Results of individual studies	19	For all outcomes, present, for each study: (a) summary statistics for each group (where appropriate) and (b) an effect estimate and its precision (e.g. confidence/credible interval), ideally using structured tables or plots.	Supplementary Tables
Results of syntheses	20a	For each synthesis, briefly summarise the characteristics and risk of bias among contributing studies.	p. 5-16
	20b	Present results of all statistical syntheses conducted. If meta-analysis was done, present for each the summary estimate and its precision (e.g. confidence/credible interval) and measures of statistical heterogeneity. If comparing groups, describe the direction of the effect.	NA
	20c	Present results of all investigations of possible causes of heterogeneity among study results.	NA
	20d	Present results of all sensitivity analyses conducted to assess the robustness of the synthesized results.	p.4
Reporting biases	21	Present assessments of risk of bias due to missing results (arising from reporting biases) for each synthesis assessed.	Limitation section
Certainty of evidence	22	Present assessments of certainty (or confidence) in the body of evidence for each outcome assessed.	NA
**DISCUSSION**			
Discussion	23a	Provide a general interpretation of the results in the context of other evidence.	p. 8-10
	23b	Discuss any limitations of the evidence included in the review.	p.8-10
	23c	Discuss any limitations of the review processes used.	p. 9
	23d	Discuss implications of the results for practice, policy, and future research.	p. 10
**OTHER INFORMATION**			
Registration and protocol	24a	Provide registration information for the review, including register name and registration number, or state that the review was not registered.	p.3
	24b	Indicate where the review protocol can be accessed, or state that a protocol was not prepared.	p.3
	24c	Describe and explain any amendments to information provided at registration or in the protocol.	p.3
Support	25	Describe sources of financial or non-financial support for the review, and the role of the funders or sponsors in the review.	NA
Competing interests	26	Declare any competing interests of review authors.	p. 10
Availability of data, code and other materials	27	Report which of the following are publicly available and where they can be found: template data collection forms; data extracted from included studies; data used for all analyses; analytic code; any other materials used in the review.	p.3-4

From: Page MJ, McKenzie JE, Bossuyt PM, Boutron I, Hoffmann TC, Mulrow CD, et al. The PRISMA 2020 statement: an updated guideline for reporting systematic reviews. BMJ 2021;372:n71. doi: 10.1136/bmj.n71

For more information, visit: http://www.prisma-statement.org/

Tab. S2. Studies included in the systematic review

SN	AUTHOR (OR FIRST AUTHOR)	YEAR	COUNTRY	GENERAL THEME	WORK FIELD	POPULATION	RESEARCH DESIGN	ANALYSIS
**1**	**Achyldurdyyeva**	2021	Taiwan	Individuals' employment environment	Finance/insurance, manufacturing, electronics, services, informatics, communication, construction, real estate, food, chemical, iron, steel	N = 2.171: all self-identified as homosexual and/or bisexual	Cross-sectional Convenience sample	Quali-quantitative
**2**	**Alden**	2020	Sweden	Sexual orientation and job satisfaction	Manufacturing, construction, trade, information, communication, transport/warehousing, finance/insurance/business, public administration, law, technology, education, health care, social services, and others (n.s.)	N = 2.504: 703 gay men, 626 lesbian women, 549 heterosexual men, 626 heterosexual women	Cross-sectional Representative sample	Quantitative
**3**	**Anand**	2022	India	Heterosexist harassment at work	Marketing, management, tourism	N = 6: 2 gay men, 4 lesbian women	Cross-sectional Convenience/purposive/theory-based sample	Qualitative
**4**	**Austin**	2021	U.S.A.	Masculine gender norms and adverse workplace safety outcomes	Manufacturing, retail, construction, finance, insurance, and others (n.s.)	N = 904: 215 homosexual, 689 heterosexual	Longitudinal Stratified sample	Quantitative
**5**	**Baker**	2017	U.S.A.	Experiences of workplace dignity	Education, mechanics, finance, waiting, hospitality, social work, amusement parks, police, restoration, law, arts, music, marketing, informatics, military forces, consulting, real estate, sales, and others (n.s.)	N = 36: 27 gay men, 9 self-identified as queer women, bisexual, lesbian woman, gay woman, queer, pansexual, transgender	Cross-sectional Convenience/purposive/theory-based sample	Qualitative
**6**	**Barnard**	2022	U.K.	Negotiation of sexual identities at work	Construction	N = 25<br>Interviews: 1 transgender man, 2 transgender women, 1 bisexual cisgender woman, 8 cisgender men self- identified as gay, and 5 cisgender women self-identified as lesbians.<br>Focus groups: 1 transgender woman, 1 bisexual cisgender woman, 5 cisgender men who identify as gay, and 1 cisgender woman self-identified as lesbian	Cross-sectional Convenience/purposive/theory-based sample	Qualitative
**7**	**Barrantes**	2018	U.S.A.	Sexual orientation and leadership suitability	Management	N = 401: all self-identified as gay, gay and out, gay and closeted, heterosexual	Cross-sectional Convenience sample	Quantitative
**8**	**Beatriz**	2022	Portugal	Employees' workplace experiences	n.s.	N = 63: 25 gay/lesbian, 17 bisexual, 4 pansexual, 1 queer, 1 asexual, 1 transgender, and 1 non-binary (28 cisgender men, 23 cisgender women, 5 non-binary, 2 transgender women)	Cross-sectional Convenience sample	Quasi-qualitative

**9**	**Blanck**	2020	U.S.A.	Employees' workplace accommodations	Law	N = 2740: 17% self-identified as LGBQ	Cross-sectional Convenience sample	Quali-quantitative
**10**	**Bonaventura**	2016	U.S.A.	Disclosure of sexual orientation at work	n.s.	N = 500: all self-identified as LGBT	Cross-sectional Convenience sample	Quantitative
**11**	**Bryson**	2017	U.K.	Wage differences	Economy (all sectors)	N = 21.981 participants (19.741 respondents): 331 heterosexual or straight, 331 gay/lesbian, 123 bisexual, 80 other, and 803 prefer not to say	Cross-sectional Representative sample	Quantitative
**12**	**Capell**	2018	EU - Israel - U.S.A. - Latin America	Trust embedded in organizational practices	n.s.	N = 431: 250 gay men, 139 lesbian women, 31 bisexual, 6 queer, and 5 transgender (258 men, 155 women, and 42 other)	Cross-sectional Convenience sample	Quantitative
**13**	**Cech**	2020	U.S.A.	Workplace inequality	Federal services	N = 330.414: 11.094 self-identified as LGBT	Cross-sectional Representative sample	Quantitative
**14**	**Cech**	2022	U.S.A.	Marginalization and devaluation at work	Science, Technology, Engineering, and Math (STEM)	N = 14.434: 594 self-identified as LGBTQ	Cross-sectional Convenience sample	Quali-quantitative
**15**	**Chen**	2020	U.S.A.	School climate and professional commitment	Psychological health care	N = 88: 19 gay men, 28 lesbian women, 26 bisexual, 2 transgender, 10 queer, and 3 questioning (19 men, 66 women)	Cross-sectional Convenience sample	Quantitative
**16**	**Clark**	2022	U.S.A.	Workplace inclusion	Federal services	N = 901.346: 28.736 self-identified as LGBT	Cross sectional Representative sample	Quantitative
**17**	**Collins**	2018	U.S.A.	Disclosure and employees' engagement	Law enforcement services	N = 12: all self-identified as gay men	Cross-sectional Convenience/purposive/theory-based sample	Qualitative
**18**	**Compton**	2016	U.S.A.	Sexual identity management at work	Sales, government, retail, chaplaincy, federal services, management, restoration, and others (n.s.)	N = 20: 8 gay men, 12 lesbian women	Cross-sectional Convenience/purposive/theory-based sample	Qualitative
**19**	**Compton**	2017	U.S.A.	Co-sexuality at work	Media, informatics, technical, education, library, management, restoration, arts, and others (n.s.)	N = 30: 15 heterosexual 7 bisexual, 4 pansexual, 2 homosexual, 1 heteroflexible, and 1 pansexual (11 male, 1 cisgender male, 12 female, 4 women, 1 transgender woman, and 1 cisgender tomboy)	Cross-sectional Convenience/purposive/theory-based sample	Qualitative
**20**	**Connell**	2021	U.K.	Career experiences	Clergy	N = 6: all self-identified as LGB	Cross-sectional Convenience/purposive/theory-based sample	Qualitative
**21**	**Corlett**	2021	Ecuador - Spain	Employees' workplace experiences	n.s.	N = 30: 14 gay men, 15 lesbian women, and 1 bisexual	Cross-sectional Convenience/purposive/theory-based sample	Qualitative
**22**	**Day**	2022	U.S.A.	Workplace bulling and PTSD	n.s.	N = 840: 155 gay men, 100 lesbian women, 57 bisexual men, 108 bisexual women, 93 heterosexual men, and 327 heterosexual women	Cross-sectional Convenience sample	Quantitative
**23**	**del Rio**	2019	U.S.A.	Occupational segregation	Law, health care, police, security services, media, art, education/training, social services, community services, human resources, social work,	N = 7.000.000: 25.874 gay men, 27.158 lesbian women	Cross-sectional Representative sample	Quantitative

					design, waiting, hairstyling, cosmetics, psychological care, science, public relations, transportation/travel, and others (n.s.)			
**24**	**Dewaele**	2019	Netherlands	Visibility management at work	Government	N = 4.239: 88.2% heterosexual, 267 (6.3%) gay/lesbian, 1.8% bisexual, 0.5% do not know, and 3.2% do not want to answer	Cross-sectional Convenience sample	Quantitative
**25**	**Dhanani**	2022	U.S.A.	Sexual identity management at work	Education, health care, retail, and others (n.s.)	N = 308: 139 (45,1%) gay/lesbian, 46,4% bisexual, 7,5% pansexual, and 1% queer (90.9% cisgender)	Cross-sectional Convenience sample	Quantitative
**26**	**Di Marco**	2018	Spain	Workplace incivility and sexual prejudices	Education, accounting/finance, civil engineering, communication, public sector, marketing/advertising, health care, charity, media/culture	N = 39: 15 gay men, 24 lesbian women	Cross-sectional Convenience/purposive/theory-based sample	Qualitative
**27**	**Donaghy**	2022	Australia	Workplace well-being	n.s.	N = 5.270: 3415 (64.8%) gay/lesbian, 21.2% bisexual, 5.8% pansexual, 4.9% queer, 2.1% asexual, 0.6% straight, and 0.5% other (42.2% cisgender men, 42.2% cisgender women, 1.5% transgender men, 2% transgender women, 3.5% non-binary, 0.5% agender, and 0.8% other gender identity)	Cross-sectional Convenience sample	Quantitative
**28**	**Einarsdóttir**	2016	U.K.	(Dis)embodied disclosure of sexual identities at work	Royal Navy, high security prison, NHS trust, international retailer, national charity, financial services	N = 50: 25 self-identified as LGB men and 25 self-identified as LGB women	Cross-sectional Convenience/purposive/theory-based sample	Qualitative
**29**	**Fahie**	2016	Ireland	Personal and professional security at work	Education	N = 23: 11 gay men, 11 lesbian women, and 1 bisexual woman	Cross-sectional Convenience/purposive/theory-based sample	Qualitative
**30**	**Federman**	2017	U.S.A.	Sexual orientation, gender identity, and perceptions of personal safety and security, job satisfaction, and diversity issues	Federal services	N = 376.577: percentage of LGBT n.s.	Cross-sectional Convenience sample	Quantitative
**31**	**Felix**	2018	Brazil	Co-construction of a climate of voice/silence in organizations	Technology, entertainment, health care, consumer products, advertising, manufacturing, banking, construction, hospitality, mining, education, transportation, utilities, chemicals	N = 65: all self-identified as gay men	Cross-sectional Convenience/purposive/theory-based sample	Qualitative
**32**	**Ferfolia**	2015	Australia	Employees' workplace experiences	Education	N = 158: 69 gay men, 89 lesbian women	Cross-sectional Convenience sample	Quali-quantitative
**33**	**Fletcher**	2021	U.K.	Supportive practice and employees' life satisfaction	Education, professional occupations, and others (n.s.)	N = 150: 90 (60%) gay men, 27 (18%) lesbian women, 9% bisexual, 8% other minority sexual identity, and 5% heterosexual	Longitudinal Convenience sample	Quantitative

**34**	**Fric**	2019	27 EU Member States - Croatia	Being out at work, discrimination, and unemployment	n.s.	N = 93.079: 59.490 gay men, 16.170 lesbian women	Cross-sectional Convenience sample	Quantitative
**35**	**Fric**	2021	Belgium - France - Germany - Ireland - Luxembourg - Netherlands - Poland - Slovenia	Employees' tenure	Elementary occupations, operators/assemblers, craft, agriculture, services/sales, clerks, technical, professionals, management, armed forces	N = 2.296.435: 9392 gay men, 8367 lesbian women	Cross-sectional Convenience sample	Quantitative
**36**	**Gacilo**	2018	North America - Western Europe - Australia - New Zealand - South Africa - Thailand - Jordan - Indonesia - Philippines	Discrimination and perceived career advantages	Media, art, security services, nurse, non-profit agencies, social work, hospitality, consulting, health care, and others (n.s.)	N = 150: 86% gay/lesbian, 11% bisexual, 3% straight, and 6% transgender	Cross-sectional Convenience sample	Quali-quantitative
**37**	**Gardner**	2022	U.S.A.	Authenticity and psychological safety at work	n.s.	N = 216: 186 (53,7%) gay men, 32 (14,8%) lesbian women, 31,5% bisexual, and 5% non-binary	Cross-sectional Convenience sample	Quantitative
**38**	**Gates**	2013	U.S.A.	Workplace stigma-related experiences	n.s.	N = 460: 215 self-identified as LGBT	Cross-sectional Convenience sample	Quantitative
**39**	**Gates**	2014	U.S.A.	Outness at work and stigma consciousness	Management, community services, social services, sales and others (n.s.)	N = 215: 120 gay men, 2 transgender, and 94 other	Cross-sectional Convenience sample	Quantitative
**40**	**Gates**	2019	U.S.A.	Sexual orientation and friendship in workplace empowerment	Social work	N = 204: 54 self-identified as LGB	Cross-sectional Convenience sample	Quantitative
**41**	**Giwa**	2022	Canada	Employees' workplace experiences	Police	N = 3: 2 lesbian women, 1 bisexual woman	Cross-sectional Convenience/purposive/theory-based sample	Qualitative
**42**	**Goetz**	2020	Germany	Outing at work	Social work	N = 189: 57 (30%) gay men, 132 (70%) lesbian women	Cross-sectional Convenience sample	Quantitative
**43**	**Green**	2021	U.S.A.	Group perception of acceptance of racial/ethnic, sexual, and gender differences	Military forces	N = 544: 248 self-identified as LGBT	Cross-sectional Convenience sample	Quantitative
**44**	**Hastings**	2021	U.S.A.	Interactional constructor of "closet"	Health care, education, social work, risk services, retail, hospitality, government, activist, banking/finance, theatre, law	N = 35: 13 gay men, 7 lesbian women, 5 bisexual, 1 bisexual queer, 2 transgender men, 2 transgender women, 1 queer, 2 queer lesbian, 1 queer gay, and 1 transgender queer	Cross-sectional Convenience/purposive/theory-based sample	Qualitative
**45**	**Hatton**	2019	U.K.	Intersectional view of the self	LGBT voluntary organizations, military forces, social work	N = 15: cisgender women self-identified as lesbian, gay, bisexual or queer	Cross-sectional Convenience/purposive/theory-based sample	Qualitative
**46**	**Henderson**	2018	U.S.A.	Sexuality-professional identity integration and leadership	Retail/sales, consulting, insurance, social work, technological support, education, and others (n.s.)	N = 135: 89 homosexual, 32 bisexual, and 12 non heterosexual	Cross-sectional Convenience sample	Quantitative
**47**	**Holman**	2019	U.S.A.	Organizational climate, support, and hostility	n.s.	N = 442 participants Subsample 1, N = 343: 108 (31.5%) gay men, 145 (42.3%) lesbian women, 15.7% bisexual, and 10.5% queer/pansexual.	Cross-sectional Convenience sample	Quantitative

						Subsample 2, N = 99: 67 (67.35 %) exclusively gay/homosexual, 25 (25.51%) predominantly gay/homosexual, and 7.14% equally gay/homosexual and straight/heterosexual		
**48**	**Holman**	2019	U.S.A.	Minority stressors at work and same-sex relationship	Social work, technical, accounting, and others (n.s.)	N = 6: all self-identified as lesbian women	Cross-sectional Convenience/purposive/theory-based sample	Qualitative
**49**	**Holman**	2022	U.S.A.	Workplace climate and identity centrality	n.s.	N = 319: 102 (32%) gay men, 134 (42%) lesbian women, 17,3% bisexual, and 9% queer or pansexual	Cross-sectional Convenience sample	Quantitative
**50**	**Hur**	2020	U.S.A.	Inclusive work practices and employees' satisfaction and commitment	Federal services	N = 421.748: 6.444 self-identified as LGBT	Cross-sectional Representative sample	Quantitative
**51**	**Jepsen**	2017	U.S.A.	Self-employment, earnings, and sexual orientation	Self-employment	N = 302.432: 67.581 gay men, 53.703 lesbian women	Cross-sectional Representative sample	Quantitative
**52**	**Jiang**	2019	China	Workplace climate, self-concealment, and self-acceptance	n.s.	N = 315s: 137 gay men, 121 lesbian women, and 57 bisexual	Cross-sectional Convenience sample	Quantitative
**53**	**Jin**	2016	U.S.A.	Employees' engagement and job satisfaction	Federal services	N = 687,687: 13.599 self-identified as LGBT, 65.562 prefer not to identify as LGBT, and 78.686 missing	Cross-sectional Representative sample	Quantitative
**54**	**Johnson**	2021	U.S.A.	Gender pronouns and organizational identity-safety cue	n.s.	N = 445<br>Study 1, N = 106: 42 homosexual, 62 bisexual, 1 pansexual, and 1queer (5 transgender)<br>Study 2, N = 172: 30 homosexual, 98 bisexual, 15 heterosexual, 14 asexual, 7 pansexual, 5 queer, and 3 other (98 transgender, 70 non-binary, 37 men, 30 women, 17 genderfluid, 14 agender, and 4 other)<br>Study 3, N = 167: 27 homosexual, 70 bisexual, 19 heterosexual, 13 asexual, 15 pansexual, 11 queer, and 12 two or more (125 transgender, 70 non-binary, 42 men, 24 women, 8 genderfluid, 12 agender, and 11 other)	Experimental design Convenience sampling	Quantitative
**55**	**Jones**	2015	U.K.	Experiences of workplace discrimination	Police	N = 836: all self-identified as LGB	Cross-sectional Representative sample	Quantitative
**56**	**Jones**	2022	U.S.A.	Employees' workplace experiences	Health care, prevention	N = 28: all self-identified as gay men	Cross-sectional Convenience/purposive/theory-based sample	Qualitative
**57**	**Kattari**	2016	U.S.A.	Housing and employment discrimination	n.s.	N = 3.838: 2.039 gay men, 1.155 lesbian women, 336 bisexual, 295 queer, 166 transgender or gender variant	Cross-sectional Representative sample	Quantitative
**58**	**Katz-Wise**	2022	U.S.A.	Experiences and perceptions of the workplace climate	Health care	N = 791: 20% self-identified as LGBQ or other	Cross-sectional Convenience sample	Quantitative

						non-heterosexual orientation		
**59**	**Kerrigan**	2020	Ireland	Sexual identity in media work	Media	N = 10: all self-identified as LGBT	Cross-sectional Convenience/purposive/theory-based sample	Qualitative
**60**	**Kim**	2019	U.S.A.	Work-Family conflict	n.s.	N = 295: 101 gay/lesbian, 144 bisexual, 17 mostly gay/lesbian, 6 other sexual orientation, 13 mostly heterosexual, and 4 asexual (179 cisgender women, 90 cisgender men, 9 transgender men, 7 gender queer, 6 transgender women, 4 other gender)	Cross-sectional Convenience sample	Quantitative
**61**	**King**	2017	U.S.A.	Employees' workplace experiences	n.s.	N = 61: 38 gay men, 17 lesbian women, 5 bisexual women, and 1 transgender bisexual woman	Longitudinal Convenience sample	Quantitative
**62**	**Klare**	2021	U.S.A.	Sexual identity, psychosocial factors, organizational differences, and intentions-to-quit	Health care, retail, sales, finance, education, government, manufacturing, transportation	N = 1.188: 1.021 heterosexuals, 167 sexual minorities	Cross-sectional Convenience sample	Quantitative
**63**	**Kollen**	2015	Germany	Management of sexual orientation at work	n.s.	N = 1.308: 471 gay men, 824 lesbian women, and 13 heterosexual and bisexual (excluded by the study)	Cross-sectional Convenience sample	Quantitative
**64**	**Kuyper**	2015	Holland	Employees' workplace experiences	Services, industry/ production, education, government, health care, welfare, and others (n.s.)	N = 9.417: 4.007 heterosexual men, 90 bisexual men, 118 gay men, 4.888 heterosexual women, 202 bisexual women, and 112 lesbian women	Cross-sectional Representative sample	Quantitative
**65**	**Laurent**	2017	France	Sexual orientation, unemployment, and participation	Industry/services, private sector,<br>public sector, and others (n.s.)	N = 106.751: 106.342 heterosexual men, 409 gay men	Cross-sectional Representative sample	Quantitative
**66**	**Lee**	2019	U.S.A.	Workplace discriminations	n.s.	N = 124: 94 (75.8%) gay men or lesbian women, 23.4% bisexual (92.7% cisgender, 1.6% transgender, and 4.8% other gender)	Cross-sectional Convenience sample	Quantitative
**67**	**Lent**	2012	U.S.A.	Sexual identity management at work	n.s.	N = 534<br>Study 1, N = 214: 165 self-identified as gay/lesbian<br>Study 2, N = 320: 142 gay men, 56 lesbian women, 88 bisexuals, and 34 pansexual (151 cisgender men, 137 cisgender women, 9 transgender men, 10 trans women, and 13 other identity)	Cross-sectional Convenience sample	Quantitative
**68**	**Lewis**	2016	Canada - U.S.A.	Labor migration and uneven landscapes of work	Social work, law, government, food services, education, non-profit agencies, scientific research, civil services, LGBT	N = 48: all self-identified as gay men	Cross-sectional Convenience/purposive/theory-based sample	Qualitative

					professional organizations, and others (n.s.)			
**69**	**Lewis**	2022	U.S:	Sexual orientation and organizational justice	Federal services	N = 42.000: all self-identified as LGBT	Cross-sectional Convenience sample	Quantitative
**70**	**Lim**	2018	Singapore	Interdependence and discrimination at work	Banking/finance/insurance, information technology, legal services, consulting, mining/oil/gas	N = 333<br>Study 1, N = 113: all self-identified as LGBT<br>Study 2, N = 220: all self-identified as LGBT	Cross-sectional Convenience sample	Quantitative
**71**	**Lloren**	2017	Swizerland	Supportive workplace polices	n.s.	N = 952: 485 gay men, 369 lesbians, 66 bisexual women, and 32 bisexual men	Cross-sectional Convenience sample	Quantitative
**72**	**Lo**	2022	China	Family and work lives	Design, information technology, media, NGOs	N = 29: all self-identified as lesbian women<br>2 lesbian chat community	Cross-sectional Convenience sample	Quali-quantitative
**73**	**Machado**	2022	Portugal	Diversity management at work	Restoration, education, engineer, administrative, health care, quality control, graphic, technical, consulting, travel, management, communication, fashion	N = 21: 12 gay men, 9 lesbian women	Cross-sectional Convenience/purposive/theory-based sample	Qualitative
**74**	**Markovic**	2022	Austria	Workplace characteristics and outness	n.s.	N = 1.177: 471 gay men, 337 lesbian women, 221 bisexual, 88 transgender and intersex, and 60 other sexual and gender identity	Cross-sectional Convenience sample	Quantitative
**75**	**Martell**	2018	U.S.A.	Employees' independence and discrimination	Management/professional, service, sales/office, construction/maintenance, production/transportation	N = 500.000: 5.598 gay men	Cross-sectional Representative sample	Quantitative
**76**	**Martinez**	2017	U.S.A.	Standing up and speaking out against prejudice	n.s.	N = 347: 80% heterosexual, 55 (16%) gay/lesbian, 3% bisexual, and 1% asexual	Cross-sectional Convenience sample	Quali-quantitative
**77**	**McFadden**	2018	Ireland	Employee's networks and stigmatization	Business, education, civil services	N = 29: 16 gay men, 11 lesbian women, 1 bisexual woman, and 1 bisexual gender queer	Cross-sectional Convenience/purposive/theory-based sample	Qualitative
**78**	**McNamara**	2021	U.S.A.	Employees' disengagement and attrition	Military forces	N = 544: 248 self-identified as LGBT	Cross-sectional Convenience sample	Quantitative
**79**	**Melton**	2014	U.S.A.	Employees' workplace experiences	Sport	N = 9: 4 gay men, 4 lesbian women, and 1 bisexual	Cross-sectional Convenience/purposive/theory-based sample	Qualitative
**80**	**Mennicke**	2018	U.S.A.	Employees' workplace experiences	Criminal justice	N = 16: 9 gay men, 7 lesbian women	Cross-sectional Convenience/purposive/theory-based sample	Qualitative
**81**	**Miao**	2021	China	Employees' workplace experiences	Social apps development	N = 32: all self-identified as gay men	Longitudinal Convenience/purposive/theory-based sample	Qualitative
**82**	**Mills**	2021	Canada	Customer abuse and aggression	n.s.	N = 723: all self-identified as LGBT	Cross-sectional Convenience sample	Quali-quantitative
**83**	**Miner**	2018	U.S.A.	Workplace heterosexism	Restoration	N = 536: 21 completely homosexual, lesbian or gay, 9 mostly homosexual lesbian or gay, and 434 completely heterosexual	Cross-sectional Convenience sample	Quantitative
**84**	**Mishel**	2020	U.S.A.	Occupational context and stereotypes	n.s.	N = 6.233: percentage of LGBT n.s.	Experimental design Convenience sample	Quantitative

**85**	**Mitchell**	2017	U.S.A.	Employees' health risks	n.s.	N = 77.968: 1952 gay/lesbian, 432 bisexual	Longitudinal Convenience sample	Quantitative
**86**	**Mizzi**	2013	Kosovo	Heteroprofessionalism at work	Humanitarian aid	N = 8: all self-identified as gay men	Cross-sectional Convenience sample	Qualitative
**87**	**Mizzi**	2022	Canada - U.S.A.	Inclusive workplace	Education	N = 15: 7 cisgender gay men, 2 cisgender lesbian women, 2 transgender queer, 1 non-binary queer, 1 cisgender pansexual man, 1 cisgender pansexual or bisexual woman, and 1 cisgender bisexual man	Cross-sectional Convenience/purposive/theory-based sample	Qualitative
**88**	**Mohr**	2019	U.S.A.	Revelation and concealment of sexual identity at work	n.s.	N = 61: 38 gay men, 17 lesbian women, and 6 bisexual	Longitudinal Convenience sample	Quantitative
**89**	**Moya**	2020	Spain	Discrimination, work stress, and psychological well-being	n.s.	N = 366: 137 heterosexual, 134 gay men, 61 lesbian women, and 34 bisexual	Cross-sectional Convenience sample	Quantitative
**90**	**Nixon**	2022	U.K.	Employees' workplace experiences	Prison officer	N = 1: self-identified as lesbian woman	Cross-sectional Single case	Qualitative
**91**	**Noronha**	2022	India	Inclusive ethical organizations	n.s.	N = 35: 24 gay men, 11 lesbian women	Cross-sectional Convenience/purposive/theory-based sample	Qualitative
**92**	**Nowack**	2020	U.S.A.	Employees' and organizational value discrepancies	Management, business, finance, and others (n.s.)	N = 180: 77% heterosexual, 14 (8%) gay/lesbian, 13% bisexual, and 2% other (53% cisgender men, 46% cisgender women, and 1% non-binary)	Cross-sectional Convenience sample	Quantitative
**93**	**O'Brien**	2020	Ireland	Roles and routines of media production	Television, film production	N = 10: all self-identified as gay/lesbian	Cross-sectional Convenience/purposive/theory-based sample	Qualitative
**94**	**Ortega**	2020	Argentina	Perceptions of anticipated stigma	Health care	N = 32: 16 gay men, 16 lesbian women	Cross-sectional Convenience sample	Quali-quantitative
**95**	**Orzechowicz**	2016	U.S.A.	Gay-friendly and post-closeted work	Park entertainment worker	n.s.	Longitudinal Single case	Qualitative
**96**	**Owens**	2022	Canada	Work-related stressors and mental health	Mining, manufacturing, transportation, agriculture, construction, finance, administration, information, management, real estate, education, health, public administration, food service, retail, arts/entertainment, and others (n.s.)	N = 531: all self-identified as LGBT	Cross-sectional Convenience sample	Quantitative
**97**	**Papadaki**	2021	Greece	Microaggressions at work	Social work	N = 10: 4 cisgender gay men, 3 cisgender lesbian women, and 3 cisgender bisexual	Cross-sectional Convenience/purposive/theory-based sample	Qualitative
**98**	**Papadaki**	2021	Greece	Employees' visibility management at work	Social work	N = 10: 4 cisgender gay men, 3 cisgender lesbian women, and 3 cisgender bisexual	Cross-sectional Convenience/purposive/theory-based sample	Qualitative
**99**	**Perales**	2022	Australia	Improvement of employees' well-being	Government, private sector and others (n.s.)	N = 31.277: 5.538 self-identified as LGBTQ+	Cross-sectional Convenience sample	Quantitative
**100**	**Periard**	2018	U.S.A.	Employee's differences at work	Federal services	N = 4029 Study 1, N = 2.014: 1917 gay men, 1462 lesbian women	Cross-sectional Convenience sample	Quali-quantitative

						Study 2, N = 2.015: 2067 gay men, 1619 lesbian women		
**101**	**Pink-Harper**	2017	U.S.A.	Employees' job satisfaction	Federal services	N = 392.752: 9.855 self-identified as LG (5.518 male, 4.054 female)	Cross-sectional Convenience sample	Quantitative
**102**	**Prati**	2014	Italy	Coming out and job satisfaction	Education, business, food services, health care, arts, travel	N = 1.460: 1.003 gay men, 343 lesbian women, 49 bisexual men, and 65 bisexual women	Cross-sectional Convenience sample	Quantitative
**103**	**Priola**	2014	Italy	Discrimination in "inclusive organizations"	Social work	N = 20: all self-identified as LGBT	Cross-sectional Convenience/purposive/theory-based sample	Qualitative
**104**	**Priola**	2018	Italy	Sexual "inclusive exclusion" at work	Social work	N = 20: all self-identified as LGBT	Cross-sectional Convenience/purposive/theory-based sample	Qualitative
**105**	**Punnakitikashem**	2019	Thailand	Supportive factors of job and life satisfaction	Operational/management in private/public/voluntary sectors	N = 144: 98 (68.1%) gay men, 25 (17.4%) lesbian women, and 14.5% bisexual (74.31 male, 25.69% female)	Cross-sectional Convenience sample	Quantitative
**106**	**Rabelo**	2014	U.S.A.	Gender harassment and heterosexist harassment	University staff	N = 212: 126 (59.7%) completely homosexual, lesbian or gay, 60 (28.4%) mostly homosexual, lesbian or gay, and 11.9% bisexual	Cross-sectional Convenience sample	Quantitative
**107**	**Rengers**	2019	Netherlands	Workplace inclusion	Humanitarian aid	N = 11: 4 gay men, 7 lesbian women	Cross-sectional Convenience/purposive/theory-based sample	Qualitative
**108**	**Rengers**	2021	Netherlands	Interpersonal antecedents of selective identity disclosure in the workplace	Logistic	N = 9: 7 gay men, 2 lesbian women	Cross-sectional Convenience/purposive/theory-based sample	Qualitative
**109**	**Rennstam**	2018	Sweden	Peripheral inclusion through informal silencing and voice	Police	N = 18: 10 gay men, 8 lesbian women	Cross-sectional Convenience/purposive/theory-based sample	Qualitative
**110**	**Resnick**	2019	U.S.A.	Microaggressions at work	Education, library, public sector, technology, health care, business, social service	N = 644: 197 (30.6%) gay men, 193 (30%) lesbian women, 16.9% queer, 10.6% bisexual, 6.7% pansexual, 1.9% heterosexual, 1.1% asexual, 0.9% fluid, 1.4% none of the above	Cross-sectional Convenience sample	Quantitative
**111**	**Rivero-Diaz**	2021	Spain	Workplace climate	Management, commerce and services, scientific/intellectual, technical, cleaning, laborer, primary sector and factory operatives, secretarial	N = 587: 291 (49.6%) gay men, 155 (26.4%) lesbian women, 23.3% bisexual, and 5.1% transgender	Cross-sectional Convenience sample	Quantitative
**112**	**Roscigno**	2019	U.S.A.	Discrimination, sexual harassment, and workplace power	Manufacturing, transportation, construction, communication, law, banking, insurance, retail, restaurants, personal services, federal and/or government	N = 6.000: percentage of LGBT n.s.	Cross-sectional Representative sample	Quantitative
**113**	**Sabharwal**	2019	U.S.A.	Employees' turnover intentions	Federal services	N = 421.748: 34.5% self-identified as sexual minorities	Cross-sectional Representative sample	Quantitative

**114**	**Sawyer**	2017	U.S.A.	Work-Family conflict	Education, business, retail, pharmaceuticals, manufacturing, management and others (n.s.)	N = 53: 14 gay men, 26 lesbian women, 11 bisexual, and 2 not identified with a category	Cross-sectional Convenience/purposive/theory-based sample	Qualitative
**115**	**Senreich**	2020	U.S.A.	Health, wellness, and employees' workplace experiences	Social work	N = 6.112: percentage of LGBT n.s.	Cross-sectional Convenience sample	Quantitative
**116**	**Singh**	2020	U.S.A.	Work stress and acceptance and commitment therapy	Arts, design, entertainment, sports, media, physical/social science, education/training/library, sales, office/administrative support	N = 8: 3 gay/lesbian, 1 pansexual, 2 asexual, 1 queer, and 1 sexually fluid (4 males,3 females, and 1 gender queer)	Cross-sectional Convenience/purposive/theory-based sample	Qualitative
**117**	**Smith**	2017	Australia	Heterosexist workplace discrimination	n.s.	N = 367: all self-identified as LGBT	Cross-sectional Convenience sample	Quantitative
**118**	**Smith**	2020	Australia	Psychological ownership and working theory	Office work, law, accounting, education, management, and others (n.s.)	N = 240: 27 (11.3%) gay men, 28 (11.7%) lesbian women, 58.3% bisexual, 9.2% queer, and 9.6% additional sexual identity not listed (25% male, 63.3% female, 20.4% cisgender, 7.1% transgender, and 9.2% agender, gender non-conforming, non-binary)	Longitudinal Convenience sample	Quantitative
**119**	**Soini**	2023	Finland	Employees' sexual identity work online	n.s.	Netnographic study on 24 discussion threads from 2 online forums (296 pages)	Cross-sectional Convenience/purposive/theory-based sample	Qualitative
**120**	**Spendler**	2023	Germany	Personality factors and disclosure of sexual orientation at work	n.s.	N = 372: 230 homosexual, 104 bisexual, 3 polysexual, 19 pansexual, 10 asexual, and 6 other (43.8% male, 52.7% female, and 3.5% other)	Cross-sectional Convenience sample	Quantitative
**121**	**Stavrou**	2021	U.K.	Employees' sexual orientation, perceived supervisory support, and job satisfaction	Private and non-profit organization	N = 21.981: percentage of LGBT n.s.	Cross-sectional Representative sample	Quantitative
**122**	**Stenger**	2018	France	Concealment and disclosure of sexual identities at work	Audit	N = 38: 12 gay men, 6 lesbian women, 11 heterosexual men, and 9 heterosexual women	Cross-sectional Convenience/purposive/theory-based sample	Qualitative
**123**	**Tatum**	2018	U.S.A.	Workplace climate and job satisfaction	n.s.	N = 214: 164 (76.6%) gay/lesbian, 15.9% bisexual, and 7.5 other (60.3% male, 36% female, 2.8% genderqueer, non-binary, etc., and 0.9% transgender)	Cross-sectional Convenience sample	Quantitative
**124**	**Thuillier**	2022	France	Perceived workplace discriminations and disclosure at work	n.s.	N = 234: percentage of LGBT n.s.	Cross-sectional Convenience sample	Quantitative
**125**	**Tilcsik**	2015	U.S.A.	Concealable stigma and occupational segregation	Psychological health care/health care, law, education, management/training, technical, informatics, mortician/funeral, arts/media, urbanistic, sociology,	N = 4.900.000: 30.343 self-identified as gay/lesbian	Cross-sectional Representative sample and convenience sample	Quantitative

					engineering, transport/travel, cosmetics/hairstyle			
**126**	**Trau**	2015	U.S.A. - Australia	Discriminatory climate perceptions, intraorganizational developmental networks, psychosocial support, and job and career attitudes	n.s.	N = 1.179: 803 (68%) gay men, 377 (32%) lesbian women	Cross-sectional Convenience sample	Quantitative
**127**	**Tsai**	2015	Taiwan	Job effectiveness and coming out	Management/supervision, manufacture, services, non-profit, and others (n.s.)	N = 319: 212 (66.5%) gay men, 107 (33.5%) lesbian women	Cross-sectional Convenience sample	Quantitative
**128**	**Tshisa**	2021	Africa	Discrimination challenges and employees' psychological well-being	n.s.	N = 9: 2 transgender women self-identified as heterosexual, 7 homosexual and/or bisexual, 5 queer, and 4 refrained from revealing their sexual identity	Cross-sectional Convenience/purposive/theory-based sample	Qualitative
**129**	**Tshisa**	2022	Africa	Employees' emotional well-being	Education, chemical, food/beverage	N = 9: 4 gay males, 1 lesbian females, 1 bisexual male, 1 bisexual female, 2 heterosexual females self-identified as transgender	Cross-sectional Convenience/purposive/theory-based sample	Qualitative
**130**	**Ueno**	2020	U.S.A.	Workplace acceptance	Transportation/communication, retail/trade, finance, services, public administration	N = 50: 16 gay men, 10 lesbian women, 7 bisexuals, 13 queer, 3 unlabeled, and 1 other (22 males, 26 females, and 1 non-binary)	Cross-sectional Convenience/purposive/theory-based sample	Qualitative
**131**	**Van Laer**	2018	Belgium	Co-workers and (homo)sexuality at work	Administrative/finance/economy, sales/commerce, travel, hairdressing, engineering, education, restoration, management	N = 31: 21 gay men, 10 lesbian women	Cross-sectional Convenience/purposive/theory-based sample	Qualitative
**132**	**Velez**	2013	U.S.A.	Workplace contexts and minority stress	n.s.	N = 326: 104 (62%) gay/lesbian, 22% bisexual, 13% mostly gay/lesbian, and 3% other minority orientation (53% women, 43% men, 2% transgender women, 2% other gender, and 0.9% transgender men)	Cross-sectional Convenience sample	Quantitative
**133**	**Viehl**	2017	U.S.A.	Employees' burnout	Mental health	N = 84: 68 (80.7%) gay/lesbian, 11.7% bisexual, and 7.6% queer (48.7% women, 42.9% men, and 8.4% transgender/queer)	Cross-sectional Convenience sample	Quantitative
**134**	**Wang**	2022	U.K.	Job-related well-being	n.s.	N = 447: 203 gay men, 125 lesbian women, and 119 bisexual	Cross-sectional Convenience sample	Quali-quantitative
**135**	**Webster**	2022	U.S.A.	Biased allocation of developmental opportunities	n.s.	N = 273: 30 (10.9%) homosexual, 10.9% preferred not to say	Cross-sectional Convenience sample	Quantitative
**136**	**Weng**	2023	U.S.A.	Geopolitical liberalism, stakeholders, and employees' firms' support	n.s.	N = 500: percentage of LGBT n.s.	Cross-sectional Convenience sample	Quantitative
**137**	**Wessell**	2017	U.S.A.	Sexual orientations disclosure and concealment at work	Education, finance, health care, services, retail, and others (n.s.)	N = 371: 125 self-identified as LGB	Cross-sectional Convenience sample	Quantitative
**138**	**Wicks**	2017	Canada	Outness at work	Education, finance, health care, armed forces, government, arts	N = 13: all self-identified as gay men	Cross-sectional Convenience/purposive/theory-based sample	Qualitative

**139**	**Williams**	2022	U.K.	Sexual orientation diversity and inclusion at work	n.s.	N = 9: 6 gay men, 1 bisexual man, and 2 bisexual women	Cross-sectional Convenience/purposive/theory-based sample	Qualitative
**140**	**Williamson**	2017	U.S.A.	Sexual orientation disclosure at work	n.s.	N = 179: 71 gay men, 108 lesbian women	Cross-sectional Convenience sample	Quantitative
